# Should Every Symptomatic Patient with Mushroom Poisoning Be Monitored in the Intensive Care Unit?—A Retrospective Observational Study

**DOI:** 10.3390/toxins18030121

**Published:** 2026-02-27

**Authors:** Duygu Kayar Calili, Demet Bolukbasi, Serife Gokbulut Bektas, Seval Izdes

**Affiliations:** 1 Department of Anesthesiology and Reanimation-Intensive Care, Faculty of Medicine, Ankara Yıldırım Beyazıt University, Ankara Bilkent City Hospital, Ankara 06800, Turkey; sevalizdes@aybu.edu.tr; 2 Department of Intensive Care, Ankara Bilkent City Hospital, Ankara 06800, Turkey; de.emeet@hotmail.com; 3 Department of Anesthesiology and Reanimation-Intensive Care, University of Health Sciences, Ankara Bilkent City Hospital, Ankara 06800, Turkey; serifegbektas@gmail.com

**Keywords:** mushroom poisoning, amatoxin, intensive care unit, acute liver failure, liver transplantation, silymarin, acetaminophen

## Abstract

A rapid method for the early diagnosis of fatal mushroom poisoning is not available. Therefore, in our region, symptomatic patients are admitted to intensive care units (ICUs) for close monitoring and treatment. This retrospective study evaluated ICU patients with mushroom poisoning to assess clinical and laboratory trends, treatment characteristics, and outcomes and determine the necessity of level 3 ICU admission. Fifty-four patients were included in this study, and the duration of ICU stay was 5.5 ± 3.9 days. Acute liver failure (ALF) was observed in 7% of patients. Eight patients (14.8%) only received symptomatic treatment, whereas two patients underwent liver transplantation, and one of whom died. The overall mortality rate was 5.6%. A significant decrease was observed in alanine aminotransferase, aspartate aminotransferase, bilirubin, urea, creatinine, lactate dehydrogenase, international normalized ratio, and prothrombin time values on the day of discharge from the ICU compared with the day of admission (*p* < 0.05). Mushroom poisoning cases thought to be at risk of developing ALF can be transferred to transplant centers early to avoid wasting time. However, to ensure that ICU resources are used effectively, we believe that monitoring and treatment in a level 3 ICU should be reserved for patients progressing to liver failure rather than all symptomatic patients.

## 1. Introduction

Wild mushroom consumption-related poisonings pose a significant health concern. The habit of gathering and consuming mushrooms from forests or meadows in Turkey, particularly prevalent in rural areas, results in a notable occurrence of mush-room poisonings, especially during the spring months. Amanita species containing amatoxin [especially *Amanita phalloides* and *Amanita Verna*] that grow in Turkey’s forests cause many serious poisoning cases every year [1]. Currently, there is no simple, rapid, accurate, and effective method for the early diagnosis of the type of mushroom ingested. For a definitive diagnosis, toxin analysis can be performed using reversed-phase high-performance liquid chromatography (RP-HPLC), or the ingested mushroom species can most likely be determined by an experienced mycologist [2,3]. However, it can often be quite difficult to access the digested mushroom, an RP-HPLC device, or an experienced mycologist.

Depending on the genus of mushroom ingested, symptoms and their onset may vary [4]. Gastrointestinal, neurogenic, and psychiatric manifestations can occur due to mushroom poisoning. Amatoxin-containing mushroom poisonings present with gastrointestinal side effects such as nausea/vomiting and abdominal pain that begin 6–24 h after ingestion and subsequently lead to acute liver and kidney failure [5,6,7]. Although clear protocols for the follow-up and treatment of patients with mushroom poisoning are lacking, the development of organ failure may necessitate intensive care management; in particular, liver failure can be fatal, especially when treatment is delayed [8,9].

In mushroom poisoning, the rapid progression of organ failure and the potential for sudden clinical deterioration make the early identification of high-risk patients and the initiation of critical care essential [5,10]. In our region, patients with mushroom poisoning showing gastrointestinal symptoms and/or abnormal transaminase values are referred to our hospital, which is a transplantation center, and these patients are directly admitted to our intensive care unit (ICU), where they are monitored and treated. Although there are a few studies evaluating mushroom poisoning cases managed in intensive care units [9,11,12], to the best of our knowledge, no study in the literature has specifically assessed patients who were directly admitted from the emergency department (ED) to a tertiary hospital’s level 3 ICU following mushroom consumption. In this context, we primarily aimed to analyze the clinical presentation, treatment approaches, temporal changes in laboratory findings, and mortality of patients with mushroom poisoning and determine the true necessity and extent of level 3 ICU admission among these patients.

## 2. Results

**Figure 1:**
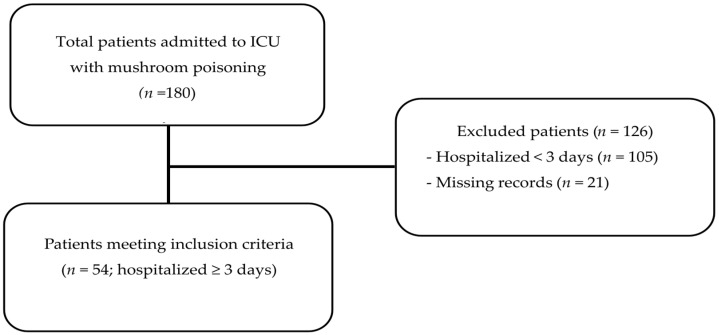
Flowchart of this study. ICU: intensive care unit.

Out of a total of 180 patients, 54 met the inclusion criteria and were thus enrolled in this study (Figure 1). The characteristics of the patients are presented in Table 1. The patients had a mean age of 55.7 ± 15.9 years, and 50% were female (*n* = 27) (Table 1). The most commonly observed comorbidities were hypertension (42.6%) and diabetes mellitus (29.6%). The duration between mushroom consumption and the onset of symptoms was found to be less than 6 h in 42.5% of the patients (*n* = 23). A patient whose symptom onset time was less than 6 h was diagnosed with ALF and underwent liver transplantation. Overall mortality was 5.6% (*n* = 3). Four patients developed ALF (7.4%), of whom two died and two were discharged, yielding a 50% mortality rate within the ALF subgroup. Among the three deceased patients, two had ALF and one had ALI. In total, 3.7% (*n* = 2) of the patients underwent liver transplantation, and one patient (50%) who underwent transplantation died. The relationship between MELDNa scores and mortality was assessed, and a significant positive correlation was observed (r = 0.395; *p* = 0.003).

The clinical and therapeutic characteristics of the patients are presented in Table 2. It was observed that 92.6% of the patients (*n* = 50) were classified as “unimpaired” according to the West Haven criteria for HE in ICU admission. Eight patients (14.8%) who were admitted to the ICU did not receive any treatment. The most frequently applied treatment was NAC treatment combined with silymarin (31.4%). NAC treatment was most frequently administered at a dose of 100 mg/kg (Table 2). When we compared the mortality rates between patients who had received the two different NAC treatment regimens, no statistically significant difference was observed (*p* = 0.501).

**Figure 2:**
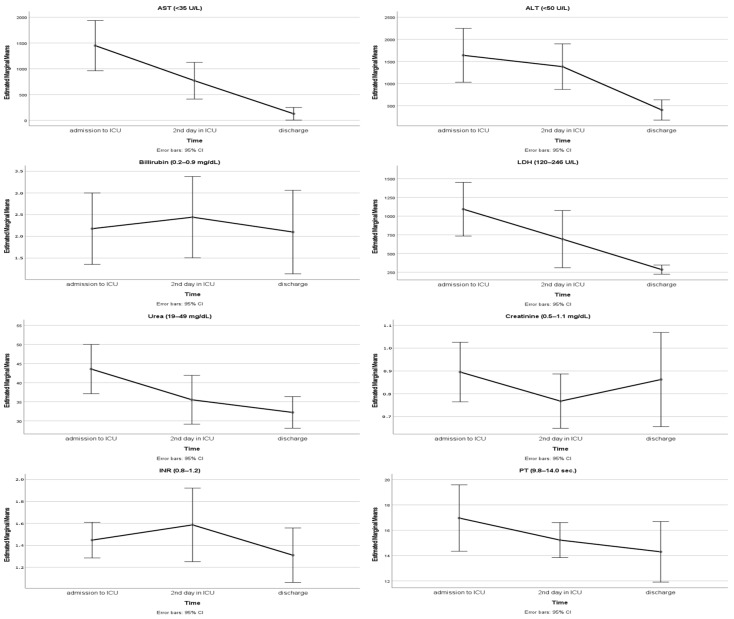
Temporal trends of laboratory parameters during ICU stay. AST: aspartate aminotransferase; ALT: alanine aminotransferase; INR: international normalized ratio; PT: prothrombin time; LDH: lactate dehydrogenase.

Patients’ peak AST values were 1516.1 ± 1737.8 U/L, and peak ALT values were 1888.8 ± 2379.3 U/L. Assessment of the relationship between peak ALT levels and length of ICU stay revealed a significant positive correlation (r = 0.383; *p* = 0.004). When the inter-temporal comparison of laboratory values was conducted, a significant decrease was observed in the ALT levels obtained before discharge compared with those obtained at admission and 48 h (*p* < 0.001) (Table 3) (Figure 2). The reduction in AST, urea, and LDH levels was significant both at 48 h and before discharge (*p* < 0.001) (Figure 2). Significant changes in bilirubin levels were observed between samples collected at 48 h and before discharge (*p* = 0.008). Significant variations in creatinine levels were observed between the admission time and blood samples taken at 48th hours (*p* = 0.001). When examining INR values, a significant decrease in laboratory levels before discharge was observed compared with blood values obtained at other time intervals (*p* = 0.004) (Figure 2). PT values were also found to be significantly decreased between admission and discharge (*p* = 0.004) (Table 3).

## 3. Discussion

In our study, the mean interval between mushroom consumption and the onset of symptoms was approximately 17 h. On average, patients were admitted to the ICU about two days after mushroom consumption, and the mean duration of ICU stay was around six days. Approximately 35% of patients had unaffected transaminase values. ALF developed in about 7% of patients; among the four patients who developed ALF, two underwent liver transplantation, and one of these patients died. The symptom onset time of this deceased patient was less than 6 h. Shock was observed in approximately 6% of patients, while about 6% had hepatic encephalopathy of grade 2 or higher. The mortality rate of mushroom poisoning monitored in our ICUs was 5.6%. All laboratory parameters showed a decreasing trend during the patients’ disease course.

Mushroom poisoning remains a significant clinical problem, particularly in regions where wild mushroom consumption is common [6,10,11,12]. It is important to take a sample of the consumed mushroom to determine whether it will cause serious poisoning. However, the type of mushroom that causes poisoning often cannot be determined. Therefore, the diagnosis and severity of mushroom poisoning are generally determined by the history of mushroom ingestion, the presence of clinical and laboratory findings related to mushroom poisoning, and the time of symptom onset. In mushroom poisoning that does not cause organ toxicity, symptoms usually manifest within the first 6 h [5,13]. The progression of poisoning is anticipated to be more benign, and gastrointestinal symptoms such as nausea, vomiting, and diarrhea; muscarinic symptoms like bradycardia and hypotension; and neurological symptoms such as euphoria, hallucinations, and somnolence may be encountered [14]. However, such non-specific gastrointestinal symptoms can likewise occur in poisonings caused by various lethal mushroom species [15]. White et al. reviewed studies on mushroom poisoning in the literature and proposed a classification based on genus, clinical profile, epidemiology, and toxicological features [16]. In their classification, mushroom poisonings were categorized into six groups: Group 1: cytotoxic; Group 2: neurotoxic; Group 3: myotoxic; Group 4: metabolic–endocrine toxicity; Group 5: gastrointestinal irritant; and Group 6: various adverse reactions to mushrooms [16]. The necessity of such a classification is emphasized for the purposes of accurate diagnosis, appropriate monitoring, and treatment planning. It is quite difficult to identify the genus of mushrooms consumed in Turkey, and we do not know the exact mushroom species responsible for the poisonings in our study cohort. All patients with a history of mushroom consumption who developed symptoms or showed laboratory abnormalities were referred for ICU admission due to concerns about potential clinical deterioration.

Symptom onset often begins 6–24 h after the consumption of *Amanita phalloides*, a highly toxic mushroom that produces amatoxins, and the onset of manifestations can extend up to several weeks [3,15,17,18]. After persisting for 12–24 h, patients may see a decline in their gastrointestinal symptoms. The period during which patients perceive themselves as having recovered is recognized as the false healing phase. However, liver failure may manifest following this period [8], and in some cases, liver failure has already started [13]. In patients with mushroom poisoning, in addition to inquiring about the type of mushroom consumed, the duration of symptoms is also specifically questioned. Pro-longed symptoms are particularly attributed to poisoning with toxic mushrooms and are associated with higher mortality [12]. In our study, the mean duration between the consumption of mushrooms and the onset of symptoms was approximately 17 h. This may be consistent with the latency period typically associated with amatoxin-containing species. Considering our patients’ mean symptom duration and even their mean time to ICU admission, we would have expected a significant portion of the patients to be in a critical state or to observe organ failure in a larger number of them. However, with this mean symptom duration, we observed ALF in only approximately 7% of the patients. On the other hand, in our study, approximately 43% of the patients experienced a symptom duration shorter than 6 h. Among patients with a symptom onset time of less than 6 h, only one patient (symptom onset time 3 h) had ALF and underwent liver transplantation; unfortunately, this patient did not survive. Liver failure was not observed in other patients with a symptom onset time of less than 6 h. Although a latency period longer than six hours is classically associated with amatoxin intoxication, early symptom onset does not necessarily exclude this diagnosis. Possible explanations include mixed mushroom ingestion, higher toxin load, or inaccurate recall of symptom timing. While severe toxicity manifestations are not commonly expected, it is deemed appropriate to monitor these patients for 2–3 days [13,14,15]. Our findings indicate that all patients should be monitored comprehensively with respect to clinical status and laboratory parameters, regardless of symptom duration. Symptom onset time alone should not be regarded as a sufficient criterion for ICU admission but rather considered in conjunction with other clinical and laboratory findings.

Mushroom poisonings may necessitate hospitalization based on the clinical condition and severity of the illness. Hospitalization durations vary depending on comorbidities, the presence of organ failure, or the development of complications, but studies indicate that hospital stays commonly range from 2 to 5 days [3,18,19]. In a study involving the evaluation of 66 patients with liver damage, the average hospitalization duration for those with liver injury was reported to be 7 days [20]. In another study including 24 patients, although the rate of patients with ALF was 4% and there were no transplantation cases, the median hospital stay was reported to be 8.5 days [21]. In our study, patients with mushroom poisoning were admitted directly to our ICU from the ED and other hospitals; patients remained in the ICU until they demonstrated clinical improvement (resolution of dehydration, cessation of vasopressor requirements, and, if present, improvement in encephalopathy) and improvement in laboratory values was observed. The mean duration of ICU stay was about 6 days. However, patients whose laboratory values did not show complete recovery or who had additional comorbidities and still required observation were transferred to the ward; therefore, the total length of hospital stay in our cohort may have been longer.

The degree of hepatic involvement via toxins can span a spectrum from normal transaminase results to the clinical picture of ALF. In the laboratory, elevation in ALT and AST levels, as well as hyperbilirubinemia due to impaired bilirubin conjugation and excretion, and an increase in INR related to decreased coagulation factor production are expected because of hepatocellular damage [3]. Additionally, an increase in urea and creatinine may be observed due to nephrotoxicity. Depending on the severity of the poisoning, liver and kidney function tests may worsen or an improvement may occur as organ function improves. Escoda et al. reported that amatoxinuria and a prothrombin rate below 83% were significant predictors of severe liver injury in mushroom poisoning, while Bernuau et al., in their editorial, highlighted additional risk factors such as female sex, pediatric age (<16 years), early diarrhea within 8 h, early ALT peak, and elevated creatinine, stressing the importance of referring such patients to intensive care or liver treatment units [22,23]. Similarly, Yang et al., in their study of 197 cases, found that patients who died had a longer symptom duration and significantly higher ALT, AST, bilirubin, creatinine, PT, and INR levels, with bilirubin being independently associated with mortality [12]. In our study, to assess changes in laboratory values, we included patients who were hospitalized for a minimum of 3 days; we evaluated their laboratory values at three different time points and analyzed their trends. Approximately 65% of patients demonstrated high transaminase values, whereas 7% had ALF at the time of admission. We think that this variability in patients’ laboratory results may reflect differences in the ingested mushroom species, toxin dose, or individual susceptibility. The moderate positive link between peak ALT levels and the length of ICU stay may indicate that the severity of liver damage reflects the overall illness. Patients with higher ALT levels needed longer intensive care, likely due to slow recovery or the need to be monitored for complications.

Increases in urea and creatinine values were also observed in some of our patients; however, there was an improvement in whole liver and kidney function tests during the ICU stay and discharge period. We believe that trends in transaminases play a crucial role in monitoring hepatic injury; however, based on our data, elevated transaminases alone do not necessarily warrant ICU admission. While absolute laboratory values provide important diagnostic information, the temporal trends of laboratory values may play a crucial role in guiding ICU admission decisions.

In patients with mushroom poisoning, rising or persistently abnormal trends in ALT, AST, bilirubin, creatinine, or INR may indicate ongoing organ injury and a higher risk of clinical deterioration, signaling the need for close monitoring in the ICU. Conversely, stable or improving laboratory trends can suggest that, despite initial abnormalities, patients may be safely monitored in level 1 ICUs or general wards with supportive care. Incorporating both clinical status and dynamic changes in laboratory values allows for a more attentive and individualized approach to ICU admission, optimizing resource use while ensuring that high-risk patients receive timely critical care.

The severity of intoxication depends on the length of time between ingestion and the beginning of treatment [18]. While standardized treatment protocols are not available, the general therapeutic principle for all mushroom poisonings is primarily symptomatic and supportive care [8]. Supportive or symptomatic treatment, such as antiemetics and hydration, typically yields favorable responses in cases of mild mushroom poisonings manifesting with gastrointestinal symptoms [24]. Gastric lavage and activated charcoal may be used to reduce amatoxin absorption from the gastrointestinal tract [25]. The drugs most often used are NAC, Pen-G, and SIL [25]: NAC is often combined with Pen-G and/or SIL [3,15], but SIL is preferred over Pen-G [8,26]. The combined treatment of NAC and SIL is reported to have a positive impact on survival [3,13]. Plasmapheresis can be employed to remove toxins in Amanita species mushroom poisonings [18]. Artificial liver support systems have limited therapeutic value, but they may be useful as bridging therapy before undergoing transplantation. Treatment in our study was tailored based on the patient’s symptoms, as mushroom identification could not be performed. We could not find any records regarding the administration of gastric lavage or activated charcoal treatment. We observed that NAC treatment was administered in 70% of the patients, with 60% receiving a combination of NAC with either Pen-G or SIL. Approximately 15% of the patients received both Pen-G and SIL treatments in conjunction with NAC. Among those undergoing NAC therapy, we noted that the treatment was administered intravenously, and two different dosing regimens were employed, namely 50 mg/kg or 100 mg/kg per day. Although different NAC regimens were utilized in our cohort, we observed no significant difference in mortality outcomes between these regimens. This variation in dosing practices likely reflects the absence of clear and standardized treatment guidelines for NAC therapy in this clinical setting.

In our cohort, 14.8% of patients did not receive specific therapies for mushroom poisoning such as NAC, Pen-G, or SIL. However, all of these patients were managed in the ICU setting and received supportive care, including intravenous hydration and symptomatic treatment. Specific therapies for mushroom poisoning were not initiated because their clinical and laboratory findings did not indicate progressive hepatotoxicity during follow-up. ICU admission in these cases was primarily precautionary, reflecting concern for potential delayed deterioration, which is characteristic of certain mushroom poisonings. As there were no standardized admission criteria during the study period, ICU hospitalization decisions were based on clinical judgment. This approach may have introduced some heterogeneity in disease severity among the admitted patients. Additionally, we posit that the inability to identify the specific mushroom species responsible for the poisoning and the heterogeneity in patients’ symptoms, clinical conditions, and laboratory results contributed to confusion among clinicians when devising treatment strategies. In our study, we observed that plasmapheresis was administered to only two patients, and none of our patients underwent artificial liver support.

After poisoning due to amatoxin-containing mushroom ingestion, fulminant liver failure may occur despite supportive treatment, and liver transplantation may be required [2,8]. There are no clear recommendations for transplantation for acute liver failure caused by mushroom poisoning. In patients with acute fulminant liver failure, a high MELDNa score predicts the need for transplantation, its timing, and the mortality associated with ALF [8,21]. In a study conducted at a transplantation center, evaluating 23 patients hospitalized with *Amanita phalloides* poisoning, it was reported that among the 6 patients who underwent transplantation, 1 had a fatal outcome [18]. In that study, when all enrolled patients were assessed, the overall survival was approximately 91%, and the rate of patients receiving vasopressor therapy was 9%. In a review assessing survival rates and transplantation outcomes in cases of mushroom poisoning, it was found that transplant recipients exhibit favorable survival rates [27]. In a study evaluating patients with acute mushroom poisoning who underwent living donor liver transplantation, those who did not undergo transplantation but received supportive treatment exhibited lower MELD scores and mortality rates compared with those who underwent transplantation [28]. In our study, approximately 7% of the patients experienced liver failure at the time of admission; the rate of receiving vasopressors was 6%; and approximately 6% of patients had grade 2 or higher HE. The mean MELDNa score of patients with ALF was <15, with transplantation performed in only two cases, one of which resulted in mortality. Higher MELDNa scores were associated with increased mortality. As MELDNa incorporates key indicators of hepatic and renal dysfunction, higher scores are likely to indicate more severe systemic involvement. This finding suggests that higher MELDNa scores may reflect not only advanced liver failure but also the presence of multiorgan involvement, which is strongly associated with an increased risk of mortality.

In our study, post-transplant mortality appears to be relatively high due to the small number of cases. Clinical evaluation of our cohort revealed that only a small number of patients required vasopressor support or exhibited signs of HE. Moreover, the limited application of advanced ICU interventions such as plasmapheresis or liver transplantation indicates that, except for patients with ALF, hemodynamic instability, or HE, routine admission to a level 3 ICU may not be necessary. These findings emphasize the importance of integrating both clinical status and laboratory results when determining the appropriate level of care for patients with mushroom poisoning. The survival rate for highly toxic mushroom poisonings is reported to be approximately 84–98% [6,12,22]. Mortality increases in cases where organ damage and ALF develop [3,18,27,29,30]. In our study, the survival rate was approximately 94%; this result may be interpreted as relatively high compared with those in other studies [6,9,12,17,20]. Several factors may explain this finding: Deaths occurred predominantly among patients who developed ALF, corresponding to a 50% mortality rate within the ALF subgroup. This finding underscores the prognostic significance of ALF in mushroom poisoning and suggests that the development of liver failure represents a critical turning point in the clinical course. The relatively favorable overall survival in our cohort may therefore reflect the limited number of patients progressing to ALF.

Direct ICU admission, even in the absence of overt organ failure, may have facilitated close monitoring and timely intervention in patients with suspected severe mushroom poisoning, particularly in those presenting with significant gastrointestinal symptoms and substantial fluid losses. Early intensive monitoring may allow for the prompt initiation of adequate hydration and supportive therapy; however, its impact on survival requires further investigation in controlled studies. This observation should not be interpreted to suggest that all patients with mushroom poisoning require level 3 ICU admission. Rather, careful risk stratification is essential to identify those who may benefit from intensive monitoring while avoiding unnecessary utilization of advanced ICU resources.

Various studies in the literature emphasize that mortality can be high among patients who develop ALF and its associated complications. Consistent with these findings, we observed that the incidence of ALF in our cohort paralleled the overall mortality rate. On the other hand, differences in the specific mushroom species ingested, toxin dose, and host susceptibility might also account for variations in mortality across different studies. The impact of the COVID-19 pandemic, which may have influenced healthcare-seeking behavior and the number of admitted cases, could have additionally affected our results. 

This study has several strengths. A notable aspect of our study is that all patients were directly admitted to a tertiary hospital level 3 ICU, which distinguishes our cohort from previous studies that have primarily included patients already hospitalized or transferred at later stages of disease progression. The inclusion of patients with a hospitalization duration of more than three days and complete clinical data ensured a well-defined cohort. Furthermore, our study comprehensively assessed clinical presentation, temporal changes in laboratory findings, treatment strategies, and outcomes, offering a broad perspective on the disease course.

However, there are some limitations to our study. The retrospective, single-center design limits generalizability and introduces potential selection bias. The overall sample size, and particularly the subgroup of patients who developed ALF or mortality, was relatively small, which may restrict the statistical power of certain analyses. As only patients with hospital stays longer than three days were included, our findings may primarily reflect cases with a sustained clinical course rather than the full spectrum of mushroom poisoning severity, potentially limiting generalizability. In addition, the exact mushroom genus responsible for intoxication could not be identified, precluding a direct correlation between clinical outcomes and specific toxins. The absence of toxicological confirmation may have led to potential misclassification of cases. Additionally, the study period coincided with the COVID-19 pandemic, during which many patients may have avoided hospital referral despite being symptomatic, potentially leading to underestimation of true mushroom poisoning cases. We also did not assess the patients’ long-term outcomes, and thus, the potential impact of mushroom poisoning on prolonged morbidity or survival remains unknown. Prospective studies on poisonings may be ethically sensitive; however, with the approval of ethics committees, non-experimental observational prospective studies that do not deviate from standard approaches and do not impose additional risk on patients may still be performed.

## 4. Conclusions

Mushroom poisonings generally result in mild clinical presentations, but they can also lead to organ failure and mortality in severe cases. Identifying the genus of mush-room that has caused the poisoning will facilitate the initiation of patient monitoring and treatment before severe toxicity and a critical clinical picture emerge. Our findings suggest that not all patients with mushroom poisoning who present with symptoms or transaminase abnormalities necessarily require admission to a level 3 ICU. However, hospitalization is essential for all such patients, ideally in units where close clinical and laboratory monitoring, especially considering changes over time, can be performed and first-line treatments can be initiated. Admission to a level 3 ICU should be reserved for those patients in whom organ failure is suspected based on their symptoms, clinical presentation, or laboratory findings. This stratified approach may optimize the use of ICU resources while ensuring that high-risk patients receive appropriate critical care in a timely manner.

## 5. Materials and Methods

### 5.1. Patient Selections

After obtaining approval from the Hospital Ethics Committee (Ankara Bilkent City Hospital; E2-23-4764), the hospital records of 180 adult patients who had been hospitalized in the ICU with a diagnosis of mushroom poisoning between March 2019 and July 2023 were retrospectively reviewed. Patient consent was not obtained due to the retrospective nature of this study. In all cases of mushroom poisoning, the diagnosis of presumed cases was based on the history of mushroom ingestion and the clinical presentation. ICU admission decisions were based on the treating physician’s clinical judgment, considering factors such as symptom severity, laboratory findings, and the potential for clinical deterioration. Patients who were hospitalized for more than three days and had no missing data were included in our study. Patients hospitalized for fewer than three days were excluded to minimize heterogeneity related to precautionary short-term ICU admissions and to allow for adequate assessment of laboratory dynamics and clinical progression.

### 5.2. Clinical Assessment and Data Collection Instruments

The demographic characteristics, time between mushroom consumption and onset of symptoms, time between mushroom consumption and admission to level 3 ICU, length of ICU stay, MELDNa (Model for End-Stage Liver Disease-Sodium) scores [28], comorbidities, patients who had undergone liver transplantation, and survival status (death or discharge) were recorded. The status of patients’ liver damage was examined in four distinct groups: those with normal transaminase values, those with abnormal transaminase values, those with acute liver injury (ALI), and those with acute liver failure (ALF). Abnormal transaminase levels were confirmed if the transaminase level was ≥10 times the normal upper limit and the INR (international normalized ratio) value was ≤1.5. Acute liver injury was defined as elevated serum transaminase levels and INR > 1.5. Acute liver failure was defined as being in an acute disease state, meeting the criteria for acute liver damage, and the presence of hepatic encephalopathy (HE).

The use of vasopressor therapy, the presence of disseminated intravascular coagulation (DIC), the presence of HE (unimpaired to grade 4; West Haven Criteria [31], administered treatments [silymarin (SIL), penicillin G (Pen-G), N-acetylcysteine (NAC) infusion duration (day) and dosage, and combinations], and whether plasmapheresis was implemented were recorded. Serum alanine aminotransferase (ALT; U/L), aspartate aminotransferase (AST; U/L), bilirubin, creatinine, urea, lactate dehydrogenase (LDH; U/L), urea (mg/dL), creatinine (mg/dL), international normalized ratio (INR), and pro-thrombin time (PT; second) values were recorded from the laboratory test results, and the timewise variation in these laboratory values was compared across different time points (admission to ICU, 48th hour, and discharge).

### 5.3. Statistical Analyses

Analysis of the data was performed using IBM version 25.0 SPSS statistics (IBM Corp., Armonk, NY, USA) and MedCalc 15.8 (MedCalc Software bvba, Ostend, Belgium) statistical software packages. Descriptive statistical methods, including frequency, percentage, mean, standard deviation, median, and min–max, were employed in the evaluation of study data. The normal distribution of the data was assessed through the Kolmogorov–Smirnov test, skewness–kurtosis measures, and graphical methods such as histograms, Q-Q plots, Stem-and-Leaf displays, and Boxplots. In cases where quantitative data did not exhibit a normal distribution, group comparisons were performed using the Kruskal–Wallis H test, and comparisons across different time points were conducted using Friedman’s test. The association between categorical variables was evaluated using the Chi-square test or Fisher’s exact test, as appropriate. Correlations between non-normally distributed continuous variables were analyzed using Spearman’s rank correlation coefficient. The statistical significance level was set at α = 0.05. Power analysis (calculated based on ALT) was conducted using G*Power 3.1.9.7 (Franz Faul, University of Kiel, Germany) statistical software. The parameters used for the analysis were as follows: *n* = 54, number of repeated measurements = 3; partial η^2^ = 0.232, effect size (f) = 0.5, α = 0.05. The determined power was found to be 99%.

## Figures and Tables

**Table 1:** Characteristics of patients with mushroom poisoning in the ICU.

		n	%
		Mean ± SD	Median (Min–Max)
Gender	Male	27	50
	Female	27	50
Age (year)		55.7 ± 15.9	59.0 (23–86)
Time between consumption and symptoms (hours)		17.1 ± 32.9	6.0 (1–168)
Time between consumption and ICU admission(days)		2.2 ± 1.7	2.0 (1–10)
Length of ICU stay (days)		5.5 ± 3.9	4.0 (2–26)
MELDNa		14.0 ± 8.4	12.5 (6–43)
Comorbidities	Hypertension	23	42.6
	Diabetes mellitus	16	29.6
	Cardiovascular D.	6	11.1
	Renal D.	3	5.6
	Respiratory D.	3	5.6
	Cerebrovascular D.	2	3.7
ICU survival	Death	3	5.6
	Discharge	51	94.4
Patients who had undergone transplantation		2	3.7
Status of liver damage	Transaminase normal	19	35.2
	Transaminase abnormal	17	31.5
	ALI	14	25.9
	ALF	4	7.4

D: disease; ICU: intensive care unit; MELDNa: The Model for End-Stage Liver Disease-Sodium; ALI: acute liver injury; ALF: acute liver failure.

**Table 2:** Patients’ clinical and therapeutic characteristics.

		n	%
		Mean ± SD	Median (Min–Max)
Patients needed vasopressor therapy		3	5.6
Patients diagnosed with DIC		4	7.4
HE	Unimpaired	50	92.6
	Grade 0–1	1	1.9
	Grade 2	2	3.7
	Grade 4	1	1.9
Therapy	None	8	14.8
	SIL	3	5.6
	Pen G	4	7.4
	NAC	4	7.4
	NAC + SIL	17	31.5
	NAC + Pen G	9	16.7
	NAC + SIL + Pen G	8	14.8
Patients who had undergone plasmapheresis		2	3.7
NAC infusion duration (days)		1.6 ± 1.2	
NAC dosage	50 mg/kg	11	20.4
	100 mg/kg	27	50

DIC: disseminated intravascular coagulation; HE: hepatic encephalopathy; NAC: N-acetylcysteine; Pen G: penicillin G; SIL: silymarin.

**Table 3:** Temporal comparisons of patients’ laboratory values.

		Mean ± SD	Median (Min–Max)
ALT (U/L)	Admission ^1^	1639.5 ± 2228.2	507.5 (10.0–8976.0)
	48th hour ^2^	1383.5 ± 1886.4	432.5 (8.0–7689.0)
	Discharge ^3^	405.3 ± 837.7	149.0 (8.0–5663.0)
	*p*	<0.001 (difference between ^3^ and ^1,2^)	
AST (U/L)	Admission ^1^	1448.9 ± 1782.9	552.5 (9.0–6369.0)
	48th hour ^2^	769.1 ± 1303.1	246.5 (10.0–5386.0)
	Discharge ^3^	128.1 ± 448.8	27.5 (7.0–3222.0)
	*p*	<0.001 (difference between ^1,2,3^)	
Bilirubin (mg/dL)	Admission ^1^	2.18 ± 3.02	1.05 (0.40–19.00)
	48th hour ^2^	2.44 ± 3.43	1.30 (0.40–18.00)
	Discharge ^3^	2.10 ± 3.53	0.90 (0.30–17.00)
	*p*	0.008 (difference between ^2^ and ^3^)	
LDH (U/L)	Admission ^1^	1093.0 ± 1314.8	517.5 (114.0–6400.0)
	48th hour ^2^	692.7 ± 1402.5	261.5 (134.0–9700.0)
	Discharge ^3^	285.1 ± 223.8	223.0 (128.0–1480.0)
	*p*	<0.001 (difference between ^1,2,3^)	
Urea (mg/dL)	Admission ^1^	43.6 ± 23.7	39.0 (0.5–122.0)
	48th hour ^2^	35.5 ± 23.4	30.0 (8.0–126.0)
	Discharge ^3^	32.2 ± 15.1	29.5 (6.0–75.0)
	*p*	<0.001 (difference between ^1^ and ^2,3^)	
Creatinine (mg/dL)	Admission ^1^	0.90 ± 0.48	0.80 (0.30–3.00)
	48th hour ^2^	0.77 ± 0.44	0.70 (0,40–3.00)
	Discharge ^3^	0.86 ± 0.76	0.72 (0.40–6.00)
	*p*	0.001 (difference between ^1^ and ^2^)	
INR	Admission ^1^	1.45 ± 0.60	1.20 (0.90–4.00)
	48th hour ^2^	1.59 ± 1.23	1.20 (0.80–9.00)
	Discharge ^3^	1.31 ± 0.91	1.10 (0.95–7.00)
	*p*	0.004 (difference between ^3^ and ^1,2^)	
PT (s)	Admission ^1^	17.0 ± 9.6	13.0 (10.0–69.0)
	48th hour ^2^	15.2 ± 5.1	13.5 (10.0–30.0)
	Discharge ^3^	14.3 ± 8.8	12.0 (11.0–70.0)
	*p*	0.004 (difference between ^3^ and ^1^)	

ALT: alanine aminotransferase; AST: aspartate aminotransferase; LDH: lactate dehydrogenase; INR: international normalized ratio; PT: prothrombin time; ^1^: indicates the time of admission, ^2^: indicates 48th hours, and ^3^: indicates the time of discharge.

## Data Availability

The data presented in this study are available on reasonable request from the corresponding author due to the legal and ethical restrictions. The authors do not have permission to share raw data.

## Abbreviations

The following abbreviations are used in this manuscript:

ICU	Intensive Care Unit
ALF	Acute Liver Failure
RP-HPLC	Reversed-Phase High-Performance Liquid Chromatography
ED	Emergency Department
MELDNa	The Model for End-Stage Liver Disease-Sodium
ALI	Acute Liver Injury
INR	International Normalized Ratio
HE	Hepatic Encephalopathy
DIC	Disseminated Intravascular Coagulation
SIL	Silymarin
Pen-G	Penicillin G
NAC	N-acetylcysteine
ALT	Alanine Aminotransferase
AST	Aspartate Aminotransferase
LDH	Lactate Dehydrogenase
PT	Prothrombin Time
